# Two jatropha karyotypes constructed from meiotic pachytene chromosomes: Pericentric distribution of heterochromatin and variation in repetitive DNAs

**DOI:** 10.1371/journal.pone.0208549

**Published:** 2018-12-06

**Authors:** Narathid Muakrong, Shinji Kikuchi, Shuto Fukuhara, Patcharin Tanya, Peerasak Srinives

**Affiliations:** 1 Faculty of Agriculture, Princess of Naradhiwas University, Narathiwat, Thailand; 2 Laboratory of Genetics and Plant Breeding, Graduate School of Horticulture, Chiba University, Matsudo, Chiba, Japan; 3 Department of Agronomy, Faculty of Agriculture at Kamphaeng Saen, Kasetsart University, Kamphaeng Saen, Nakhon Pathom, Thailand; 4 Associate Fellow of the Royal Society of Thailand, Sanam Suea Pa, Dusit, Bangkok, Thailand; Sichuan Agricultural University at Chengdu, CHINA

## Abstract

Jatropha (*Jatropha curcas*) is an oil-bearing plant used for biodiesel production. Construction of its standard karyotype and identification of the euchromatin/heterochromatin distribution associated with gene expression and meiotic recombination are essential to fully characterize its genome. Here, we developed a *J*. *curcas* karyotype based on meiotic pachytene chromosomes. In addition, a karyotype of *J*. *integerrima*, a useful species for jatropha breeding, was also constructed. Five out of eleven *J*. *curcas* chromosomes were metacentric, but only two were metacentric in *J*. *integerrima*. Almost all of the heterochromatin was distributed around the pericentric regions. The interstitial and distal regions were euchromatic without heterochromatic knobs, except for small heterochromatin regions associated with the subtelomeric repeat sequence JcSat1. These pericentric heterochromatin distribution patterns, together with chromosome structure data and the results of FISH probing with rDNA and JcSat1, allowed us to classify all chromosomes of both species. The two species had two 35S rDNA loci and one 5S rDNA locus; one 35S rDNA locus in *J*. *integerrima* was located on the interstitial region of the short arms. In addition, JcSat1 was found at only the heterochromatic ends of the *J*. *curcas* chromosome, not the *J*. *integerrima* chromosome. Despite the same chromosome number, the two pachytene chromosome-based karyotypes suggest variation in chromosome structure and distribution of repetitive DNAs in these two species.

## Introduction

*Jatropha curcas* is known as physic nut or jatropha (Euphorbiaceae) and has become one of the most important oilseed crops in the world. It is used for biodiesel production and is cultivated in areas not intended for other crops. However, genetic improvement is required for the mass production of *J*. *curcas* owing to its nondomesticated traits [1,2]. Recent studies revealed a modest level of interaccessional variability in *J*. *curcas* [3–5]; thus, the utilization of interspecific hybridization is thought to be a good option in jatropha breeding.

*Jatropha curcas* is a diploid plant with 2*n* = 2*x* = 22 [6] and a genome size of approximately 370 Mb [7]. *Jatropha* genome sequences have been previously obtained by whole-genome sequencing [8] and have been updated with newly assembled nonredundant sequences of approximately 298 Mbp from 39,277 contigs including 25,433 predicted genes [9]. Thirty-two percent of the *Jatropha* genome sequences are class I and class II transposable elements [8]. The average size of the contigs was 7,579 bp, and the N50 length was 15,950 bp [9], providing a fundamental resource for map-based cloning, resequencing and genome analysis. A syntenic relation with castor bean (*Ricinus communis*) was found on 53% of the scaffolds, suggesting a significant degree of microsynteny within the family Euphorbiaceae [8,9].

Despite the rapid progress in the genome analysis in *Jatropha*, limited success has been achieved in the cytological characterization of the *Jatropha* genomes. Using Giemsa staining, Carvalho *et al*. 2008 [7] reported the first detailed karyotype of *J*. *curcas*. Subsequently, several research groups used fluorescence *in situ* hybridization (FISH) to probe with rDNA, tandem repeat, and transposable elements to map these DNAs on mitotic metaphase chromosomes [10–12]. However, mitotic *Jatropha* chromosomes are small and similar in size, and these FISH signals can be used in the identification of a limited number of chromosomes. So far, reasonable chromosome identification has not been achieved. Although euchromatic/heterochromatic features associated with gene expression and meiotic recombination were defined at centric and telomeric regions using prometaphase *J*. *curcas* chromosomes [6], none of the karyotypes based on meiotic pachytene chromosomes (on average 10–50 times longer than mitotic metaphase chromosomes) have provided a high-resolution image. Karyotyping with pachytene chromosomes has been reported in various species, *e*.*g*., rice [13], corn [14], cotton [15] and papaya [16].

To analyze the cytological features of two *Jatropha* species, we observed well-spread meiotic pachytene chromosomes stained by DAPI and FISH signals of repetitive DNAs. Our observations revealed an overconcentration of heterochromatin at the centric region and a variation in the locations of 35S rDNA and JcSat1. These heterochromatin bands, the chromosome length, the centromere position and the distribution of repetitive DNAs were used for the identification of individual chromosomes.

## Materials and methods

### Plant materials

A plant from the local variety of *Jatropha curcas* “Chai Nat” originating from the Chai Nat province in Thailand, a commercial plant of *J*. *integerrima* with dwarf traits [17], and an interspecific F_1_ hybrid (F_1_-4) [18] derived from a cross between the two plants were used in this study. Meiotic cells were prepared from the anthers of the flower buds, whereas mitotic cells were prepared from root tips of the F_1_ plant. All three plants were grown in the field of the Department of Agronomy, Kasetsart University, Kamphaeng Saen campus, Thailand.

### Pachytene chromosome preparation

The chromosome slide preparation procedure was similar to that of Wang *et al*. 2015 [19]. Immature flower buds of 0.8–3.0 mm and root tips of 1.0–1.5 mm in length were fixed with acetic alcohol (1:3 acetic acid: ethanol) for 5 days at room temperature and then stored in 70% ethanol at 4°C until slide preparation. To prepare a meiotic slide, the flower buds were washed with Milli-Q water (MQW) for 30 min, and anthers were collected from the bud in MQW on a slide glass under a stereomicroscope. Upon this stage, slides for anthers and root tips were prepared following the same procedure. The explants were incubated with 10 μL enzyme solution (4% cellulose Onozuka RS, 2% pectinase and 1% Pectolyase Y-23) in a moist chamber at 37°C for 30 min. After maceration, the enzyme solution was removed by Kimwipe tissue paper, and a drop of 45% acetic acid was instilled. The slide was incubated for 2–5 min, covered with a cover slip and prepared according to the standard squash method.

### Fluorescence *in situ* hybridization (FISH) and genomic *in situ* hybridization (GISH)

FISH and GISH were performed using root tip samples of the F_1_ plant to observe the presence of rDNA and JcSat1 repeats on the chromosomes of each parental species. The FISH probes used were 35S rDNA (pTa71) [20], *J*. *curcas* 5S rDNA (pJc5SrDNA) [6], and JcSat1 (pJcSat1-1) [6]. 35S rDNA and JcSat1 were labeled with biotin-dUTP, while 5S rDNA was labeled with digoxigenin-dUTP using the High Prime labeling technique (Roche—Germany). For the GISH probe, the genomic DNA of *J*. *curcas* and *J*. *integerrima* was labeled with digoxigenin-dUTP and biotin-dUTP, respectively, using the High Prime technique.

The slides were incubated in 100 ng/μL RNase at 37°C for 60 min, followed by re-fixation in 1% paraformaldehyde for 10 min. Ten microliters of hybridization solution (50% formamide, 2x SSC, 10% dextran sulfate, 20 ng of each probe) was applied to each slide. For DNA denaturation, the slide was placed on a heat block at 80°C for 5 min and then incubated at 37°C for 2 days. After washing in MQW, 125 μL of detection solution (1% BSA, 4x SSC, 0.1 μg of anti-digoxigenin rhodamine (Roche) and 1 μg of FITC streptavidin conjugates (Molecular Probes)) was applied at 37°C for 60 min. The slides were washed in prewarmed MQW at 42°C for 2 min; the washing process was repeated four more times. The chromosomes were counterstained with 5 μg/mL 4,6-diamidino-2-phenylindole (DAPI) in Vectashield (Vector Laboratories—USA).

FISH and GISH signals were captured with an OLYMPUS BX-53 fluorescence microscope equipped with a CCD camera (Photometrics Cool SNAP MYO) and processed by MetaVue/MetaMorph version 7.8 and Adobe Photoshop CS3 v10.0.1.

For sequential FISH on the same chromosome samples using three probes, the coverslip was removed after the 1st detection, and the chromosome slide was washed with distilled water and dried quickly. To remove the DNA probes from the chromosomes, the slide was incubated in 50% formamide/2x SSC at 75°C for 3 min. Upon being washed and dried quickly, the slides were used for sequential FISH using the same FISH method as described above.

In the karyotype analysis, ten chromosome images were used for the measurements. The chromosome numbers from 1 to 11 of *J*. *curcas* and *J*. *integerrima* were determined by the total length of the chromosome.

## Results and discussion

### Distribution of euchromatin/heterochromatin, rDNAs, and subtelomeric repeat JcSat1 on *J*. *curcas* and *J*. *integerrima* pachytene chromosomes

**Fig 1:**
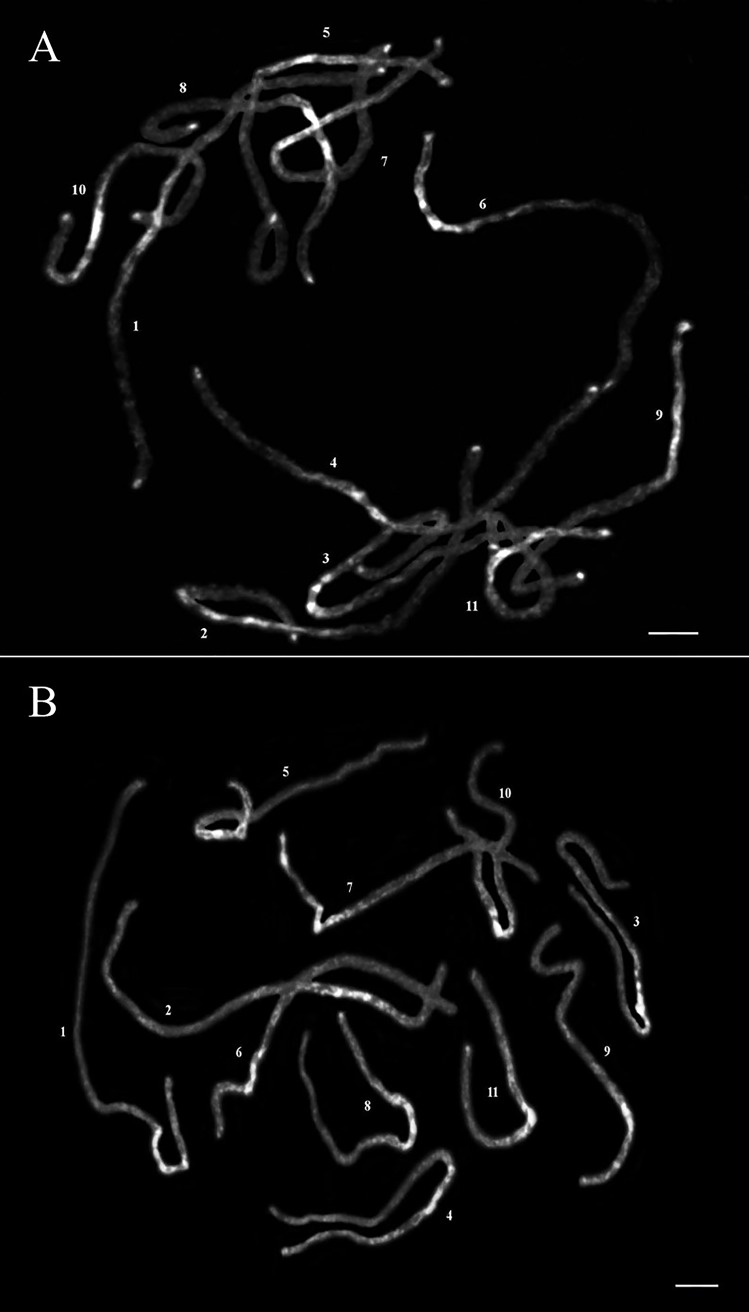
Pachytene chromosomes of *J*. *curcas* (A) and *J*. *integerrima* (B) with DAPI staining converted to a black and white image to observe distribution of euchromatin and heterochromatin on pachytene chromosomes. Bar = 5 μm.

**Fig 2:**
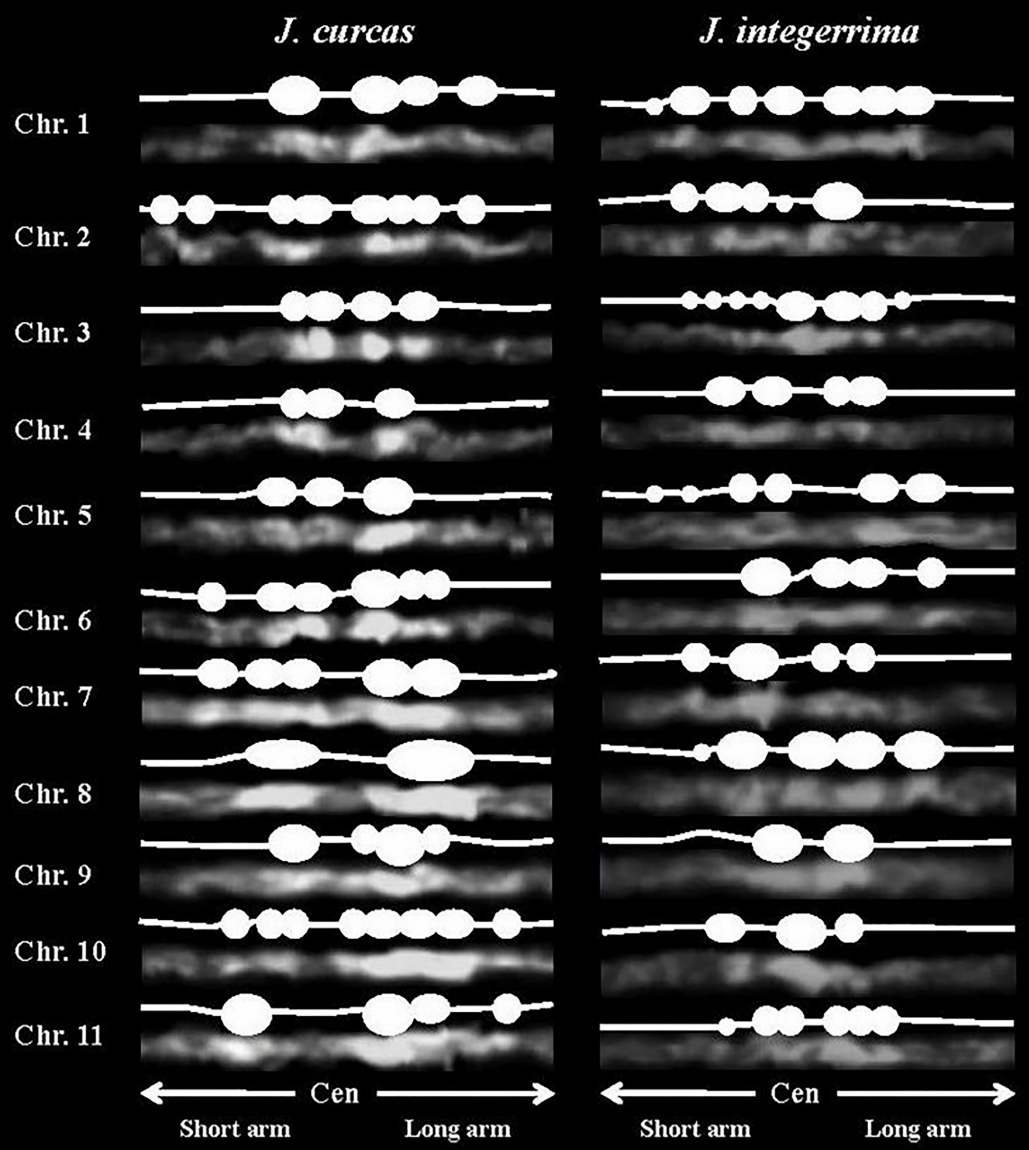
Number and locations of heterochromatin on *J*. *curcas* and *J*. *integerrima* pachytene chromosomes.

DAPI stained the heterochromatin, including AT-rich regions, more than the euchromatin. However, Marinho *et al*. 2018 [21] reported CMA^+^/DAPI^-^ pericentromeric heterochromatin (GC-rich) in all *J*. *curcas* chromosomes. To investigate in detail the distribution of heterochromatin, pachytene chromosomes longer than the mitotic chromosomes were observed. The condition and characteristics of the pachytene chromosomes depend on the pachytene stage, as estimated by the length of the flower buds. In this study, the vertical lengths of the buds were 2–2.5 mm in *J*. *curcas* and 2.5–3 mm in *J*. *integerrima*, and pachytene chromosomes with a length of 40–70 μm were suitable for observing the chromatin patterns. The overall distribution patterns of strongly stained heterochromatin on the eleven meiotic pachytene *J*. *curcas* and *J*. *integerrima* chromosomes were observed consistently at the pachytene stage (Fig 1 and Tables 1 and 2). Almost all heterochromatic knobs were distributed around the pericentric regions (Fig 1). The knobs were clearly seen, with 2–8 blocks per region (Fig 2), although some blocks were usually fused to each other at the late pachytene stage. Relatively lightly DAPI-stained chromosome arms (euchromatic regions) were observed in both species. The chromosome ends carried small heterochromatin blocks in *J*. *curcas*, but none carried small heterochromatin blocks in *J*. *integerrima* (Fig 1). Kikuchi *et al*. (2011) [6] reported a subtelomeric tandem repeat (JcSat1) associated with terminal heterochromatic blocks in *J*. *curcas*. Alipour *et al*. (2013) [12] demonstrated that *copia*-type retrotransposons produced strong FISH signals in the distal regions, similar to JcSat1. Since we could not find other heterochromatin knob structures, those repeats might be adjacent to and/or intermingled in the heterochromatin blocks.

**Table 1:** Mean ± SD for morphometric and physical location of JcSat1, 45S rDNA and 5S rDNA on J. curcas pachytene chromosomes.

Chr.	n	Short arm (μm)	Long arm (μm)	Total length (μm)	Arm ratio	RL (%)	rDNA/JcSat1	
							short arm	long arm
1	10	32.90 ± 3.50	37.34 ± 3.88	70.24 ± 5.83	1.14 ± 0.13	11.16 ± 0.52	JcSat1	JcSat1
2	10	23.33 ± 3.29	43.23 ± 4.59	66.57 ± 6.76	1.88 ± 0.24	10.55 ± 0.37	JcSat1	JcSat1
3	10	30.43 ± 3.54	33.54 ± 4.09	63.97 ± 6.59	1.11 ± 0.13	10.14 ± 0.34	JcSat1	JcSat1
4	10	26.59 ± 3.49	35.66 ± 4.35	62.26 ± 6.83	1.35 ± 0.16	9.87 ± 0.31	JcSat1	JcSat1
5	10	19.67 ± 3.42	39.38 ± 4.43	59.05 ± 6.66	2.04 ± 0.31	9.35 ± 0.26	JcSat1	JcSat1
6	10	10.72 ± 2.55	45.86 ± 5.58	56.58 ± 6.79	4.49 ± 1.09	8.96 ± 0.42	JcSat1	JcSat1
7	10	23.76 ± 4.09	31.78 ± 4.46	55.55 ± 6.92	1.36 ± 0.24	8.79 ± 0.33	JcSat1	JcSat1
8	10	23.26 ± 3.65	29.91 ± 4.09	53.17 ± 6.68	1.30 ± 0.20	8.41 ± 0.35	Jcsat1	JcSat1
9	10	15.83 ± 3.57	35.64 ± 4.54	51.48 ± 7.04	2.33 ± 0.43	8.13 ± 0.31	45S, 5S	JcSat1
10	10	16.03 ± 3.40	33.28 ± 4.65	49.31 ± 6.97	2.13 ± 0.38	7.79 ± 0.36	JcSat1	JcSat1
11	10	14.76 ± 3.26	32.39 ± 5.15	47.14 ± 7.20	2.27 ± 0.46	7.44 ± 0.34	45S	JcSat1

**Table 2:** Mean ± SD for morphometric and physical location of 45S rDNA and 5S rDNA on *J*. *integerrima* pachytene chromosomes.

Chr.	n	Short arm (μm)	Long arm (μm)	Total length (μm)	Arm ratio	RL (%)	rDNA/JcSat1	
							short arm	long arm
1	10	15.50 ± 2.86	50.95 ± 3.59	66.45 ± 5.38	3.38 ± 0.56	11.93 ± 0.27	-	-
2	10	20.29 ± 2.93	38.02 ± 4.51	58.31 ± 5.69	1.91 ± 0.33	10.47 ± 0.63	-	-
3	10	20.97 ± 4.15	36.06 ± 4.40	57.03 ± 5.46	1.81 ± 0.50	10.24 ± 0.67	-	-
4	10	23.40 ± 2.83	29.71 ± 3.23	53.11 ± 4.88	1.28 ± 0.17	9.53 ± 0.16	-	-
5	10	12.13 ± 2.91	38.42 ± 3.38	50.55 ± 5.29	3.32 ± 0.70	9.05 ± 0.15	-	-
6	10	16.59 ± 3.11	31.34 ± 3.24	47.93 ± 5.23	1.94 ± 0.34	8.58 ± 0.23	-	-
7	10	14.71 ± 2.87	32.44 ± 3.87	47.15 ± 5.54	2.27 ± 0.42	8.44 ± 0.30	45S, 5S	-
8	10	13.90 ± 2.91	32.04 ± 3.23	45.94 ± 5.07	2.38 ± 0.45	8.23 ± 0.14	-	-
9	10	15.80 ± 2.88	29.60 ± 3.34	45.40 ± 5.10	1.92 ± 0.33	8.13 ± 0.17	45S	-
10	10	14.87 ± 2.94	30.39 ± 3.42	45.26 ± 5.06	2.11 ± 0.48	8.10 ± 0.19	-	-
11	10	20.79 ± 3.27	20.04 ± 3.28	40.83 ± 5.12	0.98 ± 0.19	7.30 ± 0.45	-	-

**Fig 3:**
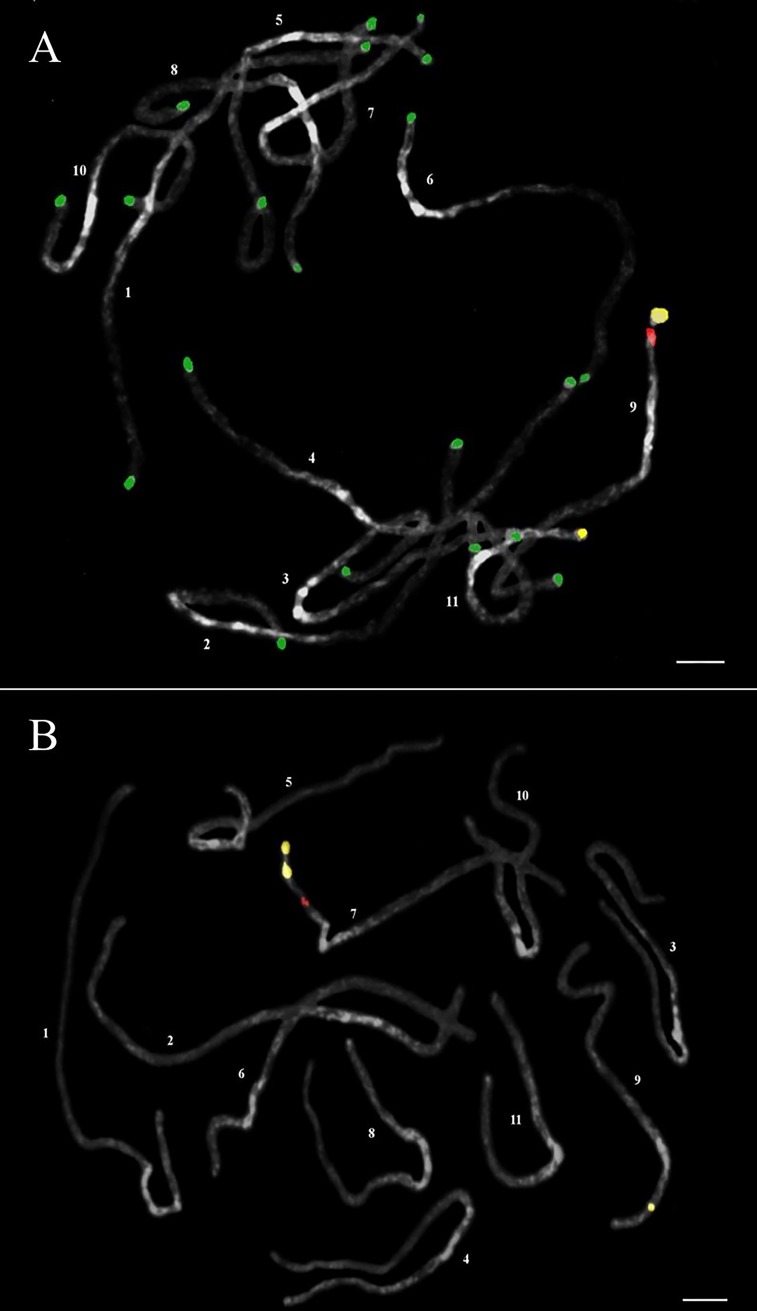
FISH signals on pachytene chromosomes of *J*. *curcas* (A) and *J*. *integerrima* (B) based on 5S rDNA (red signal), 45S rDNA (yellow signal) and JcSat1 DNA (green signal). Bar = 5 μm.

**Fig 4:**
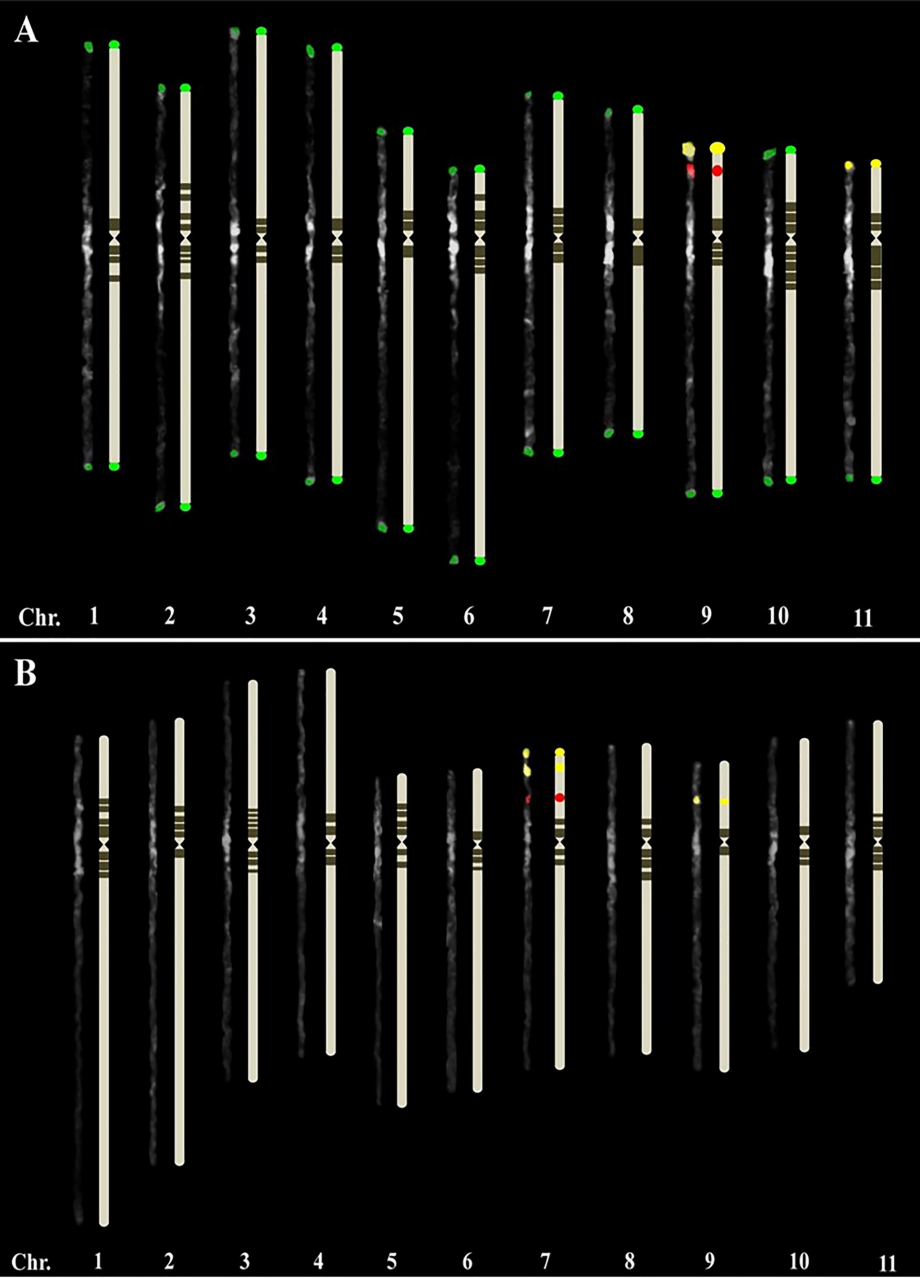
Ideogram based on pachytene chromosomes of *J*. *curcas* (A) and *J*. *integerrima* (B). Green signal is JcSat1 DNA, yellow signal is 45S rDNA, red signal is 5S rDNA, deep gray is the heterochromatin regions observed by stronger DAPI-bright region, light gray is the euchromatin regions observed by weaker DAPI-bright region.

**Fig 5:**
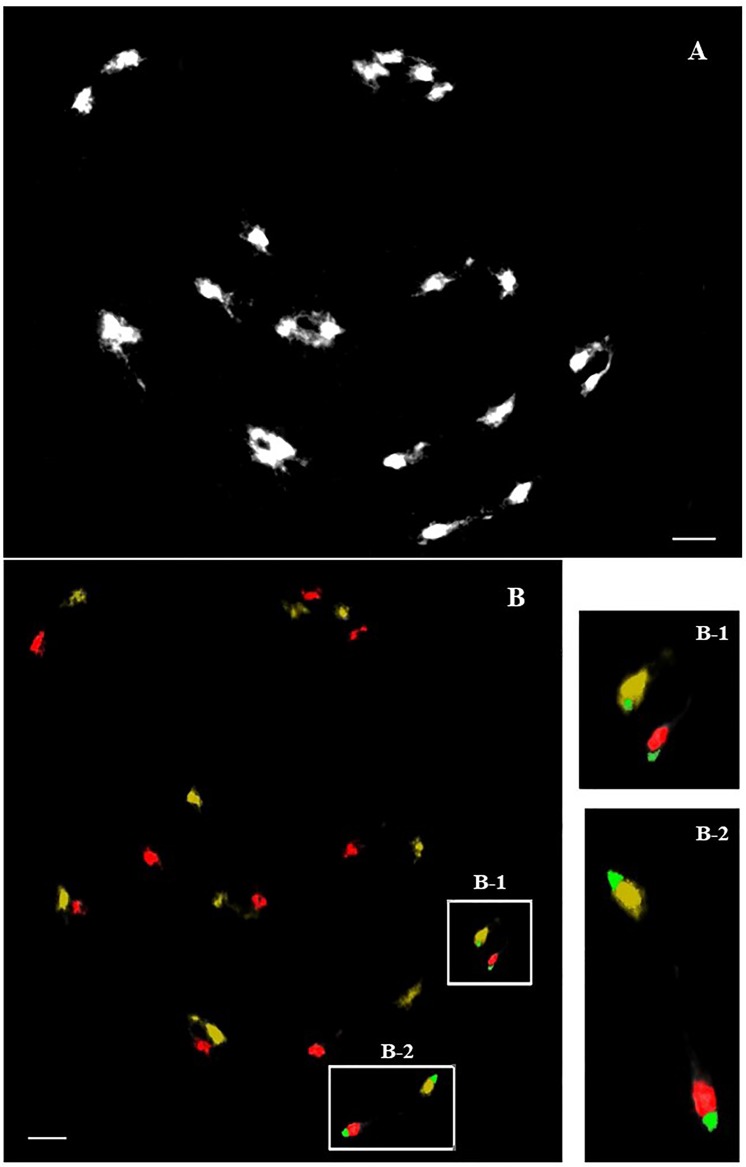
Tri-color FISH-GISH analysis on diakinesis of F_1_ hybrid. Red signal is *J*. *curcas* DNA, yellow signal is *J*. *integerrima* DNA, green is 45S rDNA signal. The weaker 45S rDNA signal (box B1) and stronger 45S rDNA signal (box B2) at diakinesis stage of the F_1_ hybrid showed homologous pairing of the chromosomes of both species. Bar = 5 μm.

A previous study reported that the chromosome pairing configuration of an F_1_ hybrid between *J*. *curcas* and *J*. *integerrima* was 0.88_I_ + 10.56_II_, and the bivalents might have formed by the pairing of homologous chromosomes from both parents [18]. However, pairs of homologous chromosomes between *J*. *curcas* and *J*. *integerrima* have not been identified so far. FISH analyses showed two 35S rDNA locus pairs and a 5S rDNA locus pair in both species (Figs 3 and 4), consistent with previous observations [21, 22]. In this study, we found that meiotic chromosomes of the F_1_ plant showed pairing between interspecific chromosomes of the same rDNA signal strength, *i*.*e*., strong *J*. *curcas* chromosome signal with strong *J*. *integerrima* signal and weak with weak (Fig 5), suggesting that the two pairs of chromosomes with rDNA are homologous. The strong 35S and 5S rDNA signals on L (linked)-type chromosomes were similar between both species. Interestingly, the S (separated)-type of the weak 35S rDNA signals were located at the interstitial regions of the short arms of *J*. *integerrima* but at the chromosome tips of *J*. *curcas* (Figs 3 and 4). FISH examination of mitotic metaphase chromosomes in five *Jatropha* species revealed that all except for *J*. *integerrima* showed terminal localization of 35S rDNA [22], although detection of a weak 35S rDNA signal at the end of the chromosomes of *J*. *integerrima* has been reported [21]. Highly resolved FISH signals using a meiotic pachytene chromosome indicated that the weak 35S rDNA signals were located at the middle of the short arms in *J*. *integerrima*. Repositioning of the rDNA foci might have occurred in *J*. *integerrima* after the divergence of the two species. Transposon-mediated rDNA repositioning events [23] and chromosome rearrangements such as translocation and inversion [24] might contribute to the position change in the 35S rDNA locus. However, the chromosome reposition pattern was not examined in this study, and only the heterochromatic pattern was observed thus far.

**Fig 6:**
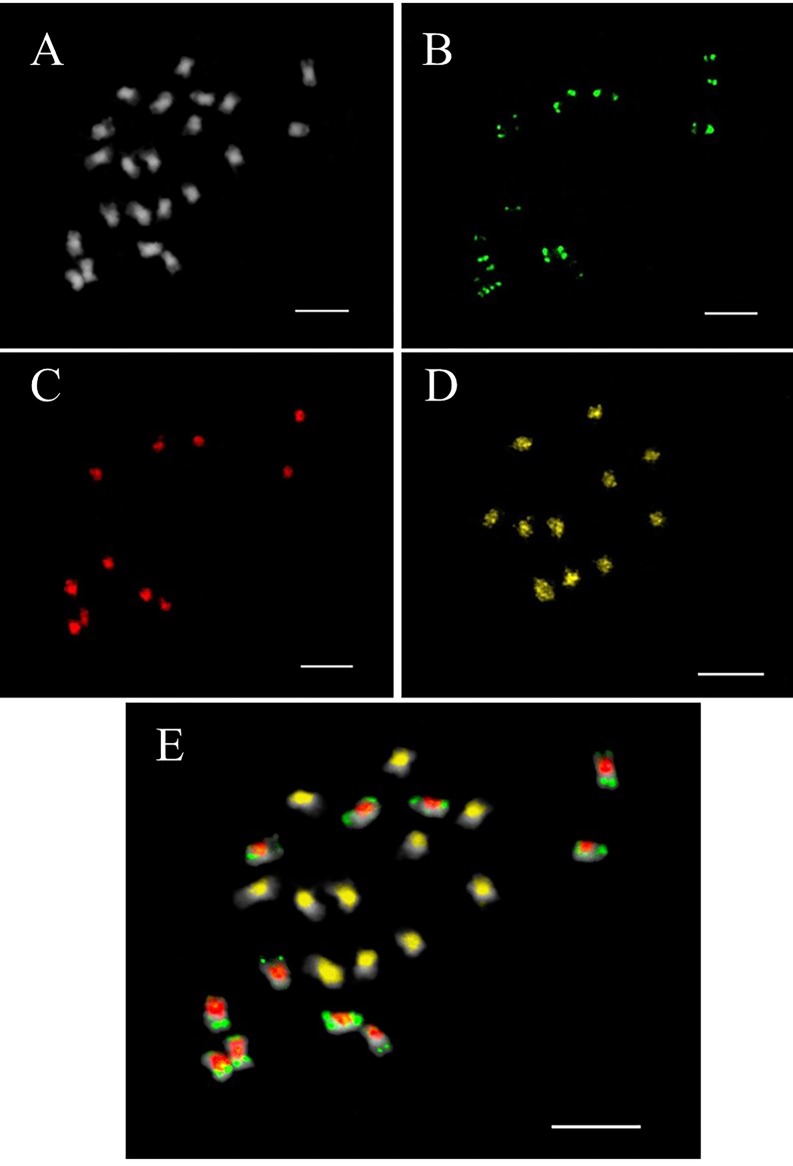
Tri-color FISH-GISH on mitotic metaphase of the F_1_ hybrid between *J*. *curcas* and *J*. *integerrima* with chromosomes 2n = 22. DAPI stained (A), green signal is JcSat1 DNA (B), red signal is *J*. *curcas* DNA (C), yellow signal is *J*. *integerrima* DNA (D). The merged image confirmed that JcSat1 DNA was specifically hybridized only on *J*. *curcas* chromosomes (E). Bar = 5 μm.

The tandem repeat in *J*. *curcas*, JcSat1, was located on the subtelomeric heterochromatin of most chromosomes but not on the chromosome ends with rDNA [6]. The currently available highly resolved FISH images also failed to reveal JcSat1 signals at the rDNA ends with long exposure times (Fig 4A). In addition, our FISH analysis revealed that *J*. *integerrima* does not have JcSat1 (Fig 4B). The *J*. *curcas*-specific detection of JcSat1 was confirmed by FISH/GISH analysis on the interspecific hybrid *J*. *curcas* x *J*. *integerrima* using meiotic cells and mitotic cells at metaphase (Fig 6). Because a close phylogenic relationship between *J*. *curcas* and *J*. *integerrima* has been suggested by phylogenic analysis based on ISSR markers [4] and by FISH analysis of the number and signal intensities of rDNA [21, 22], JcSat1 might have recently evolved in *J*. *curcas* separately from *J*. *integerrima* on the evolutionary tree.

### Karyotypes of *J*. *curcas* and *J*. *integerrima*

The length of the *J*. *curcas* mitotic metaphase chromosomes ranged from 1.71 to 1.24 μm, and Carvalho *et al*. [7] reported that five and six chromosomes were metacentric and submetacentric, respectively. For karyotyping, Kikuchi *et al*. [6] used the properties of prometaphase chromosomes in terms of chromosome structures and FISH signals of rDNAs and JcSat1 and reported a partial karyotype of *J*. *curcas* in the classification of six chromosomes that ranged in size from 6.33 to 4.04 μm. The JcSat1 signals on the pachytene chromosomes were similar in signal intensity (Fig 1), and only the rDNAs are reliable markers for *J*. *curcas* chromosomes. Although less decisive at later meiotic stages, the heterochromatic knobs in the pericentric region together with the arm ratio and total chromosome length are reliable discriminators for the identification of each chromosome pair (Figs 1 and 2 and Tables 1 and 2). In both figures, all chromosomes of the two species are arranged in order of their length.

#### J. curcas

Chromosomes 1 to 4 possess similar total lengths, and the lengths of both the short and long arms are similar, classifying these chromosomes as metacentric. Among them, chromosome 2 shows a relatively wide range of pericentric heterochromatin regions. Chromosome 3 has four pericentric heterochromatic knobs. Although chromosomes 1 and 4 are quite similar in length and arm ratio, chromosome 1 shows a third heterochromatin block on the long arm. Chromosome 5 is submetacentric, possessing light heterochromatin on the short arm side, which is thinner than that on the long arm side in the pericentric region. Chromosome 6 has the shortest short arm, with a 4.49 ratio. Chromosomes 7 and 8 are similar in length and ratio, but the large heterochromatin blocks across the centromere on chromosome 8 are useful discriminators. Classification of chromosomes 9 to 11 can be achieved by observing the locations of the JcSat1 and rDNAs signals on their arms (Table 1).

#### J. integerrima

Chromosome 1 is the longest subtelocentric-like chromosome, with a 3.38 arm ratio (Table 2). Distinguishing chromosomes 2 and 3 may be a challenge due to the similar total chromosome length and arm ratio. Chromosome 2 possesses small heterochromatin blocks on half of the short arm. However, depending on the chromatin condensation, chromosome 3 shows a relatively longer short arm and appears nearly metacentric. Chromosomes 4 and 11 are metacentric with different lengths. Pericentric heterochromatin on chromosome 5 covers half of the short arm; this chromosome is subtelocentric. Chromosomes 6, 8 and 10 display similar length and arm ratios. However, a distant pericentric heterochromatin block on the long arm side of chromosome 6 and four pericentric heterochromatic knobs on chromosome 8 can help distinguish the three chromosomes. Chromosomes 7 and 9 can be distinguished by the presence of stronger rDNA signals on chromosome 7 (Table 2).

In conclusion, the constructions of the karyotypes provide knowledge of overall chromosomal features, *i*.*e*., the length of each chromosome, chromatin condensation, and distribution of repetitive DNAs. Although less decisive at later meiotic stages, each pachytene chromosome is distinguishable. In a future study, FISH-based karyotypes with single-copy genes [25] and bulked-oligonucleotide probes [26] will be used as tools for karyotyping pachytene chromosomes to obtain an even higher resolution.
